# Topics of Public Interest

**Published:** 1905-06

**Authors:** 


### TOPICS OF PUBLIC INTEREST.

### Defeat of the Osteopathic Bills in New York and Pennsylvania.

PENNSYLVANIA.

THE osteopaths, through well organised effort, have appealed to a number of state legislatures for recognition. Bills almost identical in verbiage were introduced in several of the state legislative bodies, the purport of which was to confer upon the persons who practice osteopathy the same privileges as are accorded "other physicians" under the public health laws, and the creation of separate state boards of osteopathic examiners.

In Pennsylvania the bill, modified in some particulars, passed both houses of the legislature and went to the governor for his action. Governor Pennypacker, after hearing both sides and giving the matter mature deliberation, vetoed the bill, giving his reasons therefore in the following language:

I file, herewith, in the office of the secretary of the commonwealth, with my objections, Senate Bill No. 115, entitled "An Act to regulate the practice of and licensing of osteopaths in the state of Pennsylvania, the establishment of a board of osteopathic examiners representing the Pennsylvania Osteopathic Association and providing for the punishment of persons violating the provisions of this act."

The purpose of this bill is to establish a state board of osteopathic examiners, to consist of five members to be appointed by the governor. This board is directed to issue certificates of qualification to practise osteopathy to all applicants having a diploma from a legally recognised and regularly incorporated college of osteopathy. It further provides that licenses shall be issued to all persons who have practised osteopathy in the state for four years prior to July 1, 1905, and to such persons as now legally hold a diploma from a reputable and legally conducted college of osteopathy. It further provides that the applicants after July 1, 1905, shall have attended for at least three separate years such a college of osteopathy and shall be examined by the board in certain designated branches, and that those who apply after July 1, 1907, shall in like manner have studied for four separate years and submit to such examination.

Section 7 provides that "any person who shall practise or attempt to practise osteopathy in treating diseases * * * without having first obtained the license herein provided for * * * shall be deemed guilty of a misdemeanor and upon conviction thereof shall be punished by a fine of not more than one hundred dollars or by imprisonment in the county jail for a period of not more than ninety days for each offense."

There is nothing in the bill to indicate what constitutes the science of osteopathy. An effort to solve the query by an examination of the printed literature of the science is not very successful. Dr. A. T. Still, who is the founder of the science and is still living, in his autobiography, says, "Who discovered osteopathy? Twenty-four years ago, the 22d day of next June, at ten o'clock, I saw a small light in the horizon of truth. It was put in my hand, as I understood it, by the God of nature. That light bore on its face the inscription: 'This is my medical library, surgery and obstetrics. This is my book with all directions, instructions, doses, sizes, and quantities to be used in all cases of sickness, and birth, the beginning of man: in childhood, youth and declining days.' I am something of what people call 'inspired.' * * * The other classes have different names for it—clairvoyance and clairaudience. Sometimes I was so clairvoyant that I could see my father twenty miles from home: I could see him very plainly cutting a switch for my brother Jim and

I, if we hadn't done a good day's work. That is called clairvoyance. Then I could hear him say: 'If you don't plow faster I will tan you twice a week.' That is clairaudience." In his Philosophy of Osteopathy, he says: "Principles to an osteopath means a perfect plan and specification to build in form a house, an engine, a man, a world, or anything for an object or purpose. To comprehend this engine of life of man which is so constructed with all conveniences for which it was made, it is necessary to constantly keep the plan and specification before the mind, and in the mind, to such a degree that there is no lack of knowledge of the bearings and uses of all parts."

However true this may be, it is too indefinite to give the ordinary layman any very accurate description of the science. Since the bill refers to other schools for the practice of medicine, it is probably a school and it appears from section five that the holders of certificates are not to prescribe or use drugs or operative surgery. The main reliance would appear to be upon the manipulation of muscles and nerves, but this conclusion is entirely an inference and may be incorrect. It must be assumed, however, that there is some scientific truth which those interested in osteopathy have discovered and that it is utilised in the treatment of diseases. The study of medicine and surgery has been conducted for ages, and we have much legislation which is applicable to its conduct. Under the act of May 18, 1893, a medical council was established which issues licenses after examinations, authorising the applicants to practise medicine and surgery. If then osteopathy represents some truth in the treatment of disease, the knowledge and use of which would be beneficial to the diseased and the injured, with what consistency can these practitioners be prevented from making such use of it? Why should their patients be deprived of such benefit as may result from the discovery? If, however, this bill should become a law, the physician who would practise or attempt to practise what the osteopaths have discovered would be punishable by imprisonment for ninety days. This would be possibly a great loss to the community and certainly a great wrong to the physicians. The whole thought of the establishment of "schools" of medicine is unscientific. All of those engaged in such pursuit ought to be seeking to ascertain the truth and to accept it wherever it may be found. The approval of this bill would appear to give the authority of the state to a system of practice in the healing art which excludes the use of medicine and the use of surgery. Why should there be an attempt to so confine the operations of the mind? If both drugs and surgery are useless they may be rejected, but if they should at times be found to be beneficial why should any science for the enforcement of a theory make the effort to exclude their use?

Should the bill become a law licenses would be issued by the state board of osteopathic examiners and not by the medical council of Pennsylvania, which would be an anomaly in our legislation upon the subject. For these reasons the bill is not approved.

We take pleasure in printing the text of Governor Pennypacker's veto in full, for the reason that it is a model of clearness and betrays the proper conception of the relationship between a state executive and the medical practice laws. It is a pity the governors of some other states do not have the same respect for the statutes regulating the practice of medicine that Governor Pennypacker possesses.

### NEW YORK.

In the state of New York the campaign was waged by the osteopaths much on the same lines as in Pennsylvania and other states. It is probable that they put up the best fight that was in them. They were fortunate in having secured one of the older senators, speaking from the standpoint of senatorial service, to introduce their bill and to champion it through the several stages of committee action up to final vote in the senate.

It seems almost inconceivable that such a monstrous measure should ever endanger the fair name and fame of the Empire State; but it came within two votes of passing the upper house. The senate is composed of 50 members and under its rules 26 votes are required to pass a bill. This mongrel, in expression and meaning, was put upon its final passage April 25, 1905, at which time it received 24 votes. The *Evening Sun* (New York) of that date published the following despatch which we print in full, including the headings:

### OSTEOPATHISTS LOSE.

#### SENATE KILLS THE BILL GIVING THEM RECOGNITION.

Albany, April 25.—Senator Davis's bill, placing osteopathy under the jurisdiction of the state board of regents and compelling all masseurs in this state to pass an examination, was defeated in the senate today by a vote of 24 ayes to 19 noes, two less than the number required to pass the bill. The osteopathists have been trying to get legislative recognition for some years and came nearer this year than ever before.

The vote was as follows:

Aye—Messrs. Ambler, Brackett, Burr, Cassidy, Davis, Dooling, Fechter, Fitzgerald, Frawley, Gardner, Gates, Goodsell, Grady, Hasenflug, Hawkins, Hinman, Keenan, Kehoe, L'Hommedieu, McCarren, Raines, Riordan, Tully, and Wilcox—24.

Nay—Messrs. Armstrong, Brown, Carpenter, Coggeshall, Cordts, Cullen, Drescher, Fancher, Foley, Lewis, Malby, Marks, Martin, McEwan, Page, Saxe, Warnick, and White—19.

Absentees were Allds, Barnes, Cooper, Elsberg, Hill, Prime, and Stevens.

Another effort will be made to pass the bill.

We are unable to learn that the senior senator from Erie county, notwithstanding the threat implied in the last paragraph of the *Sun's* despatch, ever made a second attempt to pass the bill. To the legislative committee of the medical society of the state of New York, supported by similar committees representing the other state societies and the county auxiliaries, the medical profession of the Empire State is indebted for this escape from the worst blot on its escutcheon that has ever threatened it.

Senator Horace White, of Syracuse, led the opposition to the bill on the floor of the senate on the day in question in a masterful manner, and to his skill in debate and parliamentary practice, we owe, in large part, the defeat of the bill in the senate, which in reality settled its legislative status.

---

### Fever at Panama.

THAT there is some yellow fever at Panama is not surprising. It would, all things considered, be surprising if there were none. Down to a year ago the sanitary state of the isthmus was simply appalling. The wonder is that the whole place was not more plague scourged than it was. To make it quite healthful within a single year would, in the most favorable circumstances, have been a remarkable achievement. But the circumstances have not been most favorable. In some respects they have been unfavorable. We have already commented upon the report upon the subject which was made by Dr. C. A. L. Reed. Injudicious and intemperate as was that document in some respects, it contained much truth and set forth convincingly the unwarranted difficulties with which our heroic health officers on the isthmus have had to contend. . . . . .

That there is some yellow fever down there is not discouraging, either. Despite all the delay, our administrators are making progress in the introduction of water into the cities and the creation of sewers and the paving of the streets. Before the end of this year such works will be done. Then we may confidently expect marked improvement in health conditions. The reorganisation of the canal commission will unquestionably result in expedition of the work and in the giving to the health officers a freer hand in the prosecution of their labors. People ask why Colonel Gorgas could not have eradicated yellow fever there as promptly as he did at Havana. The answer is that he has not had at Panama the free hand that he had in Cuba. Besides, Panama is much further south than Havana, and some of its natural and acquired conditions are much worse. Nevertheless, give him a fair chance—and we shall see what we shall see.—*The Tribune.*

